# Aortic stenosis: what is the role of aging processes?

**DOI:** 10.18632/aging.101826

**Published:** 2019-02-11

**Authors:** Nancy Côté, Philippe Pibarot, Marie-Annick Clavel

**Affiliations:** 1 Institut Universitaire de Cardiologie et de Pneumologie de Québec/Quebec Heart and Lung Institute, Department of Medicine, Laval University, Quebec, Canada

**Keywords:** aortic stenosis, pathophysiology, aging, calcification, pathways

Calcified aortic stenosis (AS) is the most frequent valvular heart disease in high-income countries. AS is yearly responsible for approximately 20,000 deaths and 1000,000 aortic valve replacements, in North America, which individually costs 120,000$ making this disease a huge health and socio-economic burden. For a long time, AS has been considered as a passive degenerative disease resulting from aging and ‘’wear and tear’’ of the aortic valve. This perception has changed over the years with the publication of several studies showing that the aortic valve sclerosis shares many cellular similarities with vascular atherosclerosis, and supporting the concept that AS is an active disease. Furthermore, AS has been linked to several traditional risk factors for coronary artery disease, including: age, male sex, hypercholesterolemia, diabetes, hypertension, smoking, and obesity. This raises the possibility that AS might be a modifiable disease. However, the treatment to substantially slow the progression of AS and then, avoid thousands of valve replacements and save lives every year, is not yet available.

**Figure 1:**
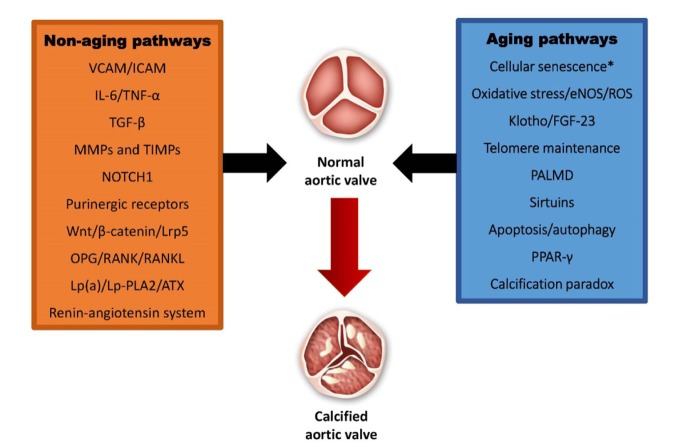
**Non-aging and aging pathways involved in calcified aortic stenosis.** VCAM: vascular cell adhesion protein; ICAM: intercellular adhesion molecule; IL-6: interleukin-6; TNF-α: tumor necrosis factor alpha; TGF-β: transforming growth factor beta; MMPs: matrixmetalloproteinases; TIMPs: tissue inhibitor of metalloproteinases; Lrp5: low-density lipoprotein receptor-related protein 5; OPG: osteoprotegerin: RANK: receptor activator of nuclear factor kappa-B; RANKL: receptor activator of nuclear factor kappa-B ligand; Lp(a): lipoprotein(a); Lp-PLA2: lipoprotein-associated phospholipase A2; ATX: ataxin; eNOS; endothelial nitric oxide synthase; ROS: reactive oxygen species; FGF-23: fibroblast growth factor 23; PALMD: palmdelphin; PPAR-γ: peroxisome proliferator-activated repector gamma

Many "non-aging" cellular pathways such as NOTCH1, Wnt-β catenin/Lrp5, OPG/RANK/RANKL, the renin-angiotensin system, and pro-inflammatory pathways including IL-6, TNF-α, and TGF-β have been implicated in inflammation, calcification, and fibrosis of the aortic valve (Figure 1). However, it is well known that the incidence of AS increase with age and recently, many possible aging pathways involved in the initiation and progression of AS have been discovered leading to a new era of therapeutic strategies (Figure 1).

Constant haemodynamic stress or common AS risk factors can induce valvular endothelial cell denudation. The aortic valve is a predilection site for endothelial turnover by its chronic exposure to high levels of mechanical stress. Under pathological conditions related to biological "aging", endothelial cell denudation is no longer replaced by circulating endothelial progenitor cells and senescent endothelial cells are accumulating on the surface of the aortic valve. These processes may lead to advanced aortic valve lesions, modification of gene expression profile and open the way to lipid infiltration. Chronological aging endothelial cell senescence is accompanying by increased reactive oxygen species, inflammatory response and reduced eNOS/NO level [1]. These modifications in concert with dysregulation of antioxidant mechanisms by elevated levels of H_2_O_2_, superoxide, and reduced levels of superoxide dismutase can contribute to increased oxidative stress of infiltrated lipids, which in turn, can activate deleterious pro-inflammatory mechanisms in the aortic valve [2].

Sirt1, an anti-aging and anti-inflammatory protein, has been shown to inhibit the expression of the pro-inflammatory cytokine resistin in AS. Sirt1 level is reduced in explanted valves from AS patients and is negatively associated with the expression of resistin [3]. Noteworthy, in AS patients operated for severe AS the main findings are that elderly patients have a less pro-atherogenic plasma lipid profile whereas their circulating resistin levels are increased compared to younger patients [4]. Interestingly, higher resistin level has been associated with increased valvular inflammation and calcification in elderly patients [4]. However, these two mechanisms have never been studied in AS. Sirtuins are suspected to control mitochondrial biogenesis and reduce oxidative stress, again, acting as anti-aging agents [5]. All together, these findings suggest that age-related modification of the adipokine system might play a role in the development of AS in the elderly population.

The extensively studied Klotho mutant mouse presents not only phosphate retention but also a premature syndrome resembling human aging. Klotho-deficient mice are characterized by multiple aging-like pathologies such as endothelial dysfunction, soft tissue calcification, atherosclerosis, and shortened lifespan. Klotho is not only expressed by cells, but can be found as a secreted protein, which level declines with the normal aging process [6]. Although the serum level of Klotho in AS patients remains to be determined, in a small sample study, Klotho was lower in excised calcified aortic valve when compared to control aortic valves. Moreover, Klotho gene deficiency in a mice model promotes aortic valve fibrosis through AMPKα-mediated activation of pro-calcifying and fibrotic gene RUNX2 [7]. On the other hand, restoration of serum Klotho level in senescence-accelerated mice P1, an aging model characterized by fibrotic aortic valve disease, effectively suppresses inflammation and attenuates aortic valve fibrosis [6].

Another, important marker of aging is the individual variations of telomere length that are affected by genetic and non-genetic factors. The human telomerase is responsible for maintaining and elongating telomeres. Critically short telomeres lead to cellular senescence and dysfunction, which in turn reduce the repairing and regenerative capacities of the cardiovascular system. In AS patients, leukocytes telomeres length is shorter than in healthy matched patients and telomerase activity is yet to be elucidated [8]. In an interesting recent animal model, telomeres shortening in NOTCH1-haploinsufficient mice is sufficient to elicit age-dependant premature calcification of the aortic valve via RUNX2 pathway (Theodoris CV et al. 2017).

In the last decades, obvious differences between young and old AS patients have been cast to light. The insulin-resistant state and atherogenic dyslipidemia related to visceral obesity may have a predominant role in the development of aortic valve inflammation and calcification in the younger population. Although, other mechanisms may be involved in the older population, including dysregulation in the mineral metabolism, postmenopausal deficiency in estrogen, and age-related modification in the adipokine system. More research is crucial to better characterize the potential aging mechanisms implicated in the development and progress of AS. Such research may help to tailor new anti-aging therapies targeting AS in older patients or in patients with accelerated aging process.
